# Atlanta Academy of Medicine

**Published:** 1874-11

**Authors:** 


### Atlanta Academy of Medicine.

E. L. CONNALLY, M.D., Reporter.

Atlanta, October 5, 1874.

Dr. Woodhull, U. S. A., reported a case of epilepsy in an officer, reading notes of three severe seizures that he had had during the past four months, each growing lighter. During September he had been absent in apparent good health. About 10 P.M. of the 4th instant, while about to take the cars at the depot, he fell, without a cry or apparent warning, in a convulsion, and with a seizure of unconsciousnes from which he never rallied. It became apparent that he would not speedily pass out of the fit, as he had previously done. Dr. Battey was called.

Dr. Battey: Found him breathing very heavily; hands clenched; rose-colored froth passing from his nose and mouth; carried him into the saloon and proceeded to apply sinapisms to chest, spine and extremities; pulse 50, small, soon increased to 80, and then went back to 50; respiration quite labored. In the course of half hour Dr. Woodhull reached the patient, and they then ordered foot-baths of scalding water; says he meant scalding when he said so, for he had found a foot-bath of that temperature of great service in rousing up comatose patients; ordered enemata of tr. capsicum and water; found the sphincter contracted and of good tone; had seen patients in similar condition with the sphincter usually very much relaxed. There was no return of consciousness.

Dr. Battey says this case reminded him of several cases he had seen of gastro-intestinal disorder, resulting in coma; but there was here no history of imprudence in diet.

Dr. Woodhull continued the report of the officer's case, by saying that about 1 o'clock, with Dr. Battey's concurrence, he removed him to his quarters at the barracks. He evinced no evidence of consciousness, and died at 4 o'clock in the morning.

Dr. Battey thinks that the mass of the profession are not sufficiently energetic in their treatment of such cases. If we are to bring to life these extreme cases, we must be *in earnest.* Scalding foot-baths and enemata of tr. capsicum, he has found to be very useful; not much risk to run; if we can swap a fatal coma for a diarrhœa, or a scalded foot, thinks it a good exchange.

Dr. J. G. Westmoreland thought there were different causes operating in the production of coma, and did not think one treatment would do for them all.

Dr. W. F. Westmoreland asked Dr. Battey if he would treat an opium coma as he did the above case?

Dr. Battey thinks the first thing to be done is to wake them up, and the above treatment he found the best.

Dr. W. F. Westmoreland had seen cases of hysteria with coma, and did not think the above treatment appropriate in such cases.

Dr. Battey—If he did not have the intelligence to discriminate, he would *wake them up.*

Dr. W. F. Westmoreland thought there were cases of coma where the safety of the patient depended on the continuance of the coma for a time.

Dr. Harden called the attention of the Academy to an epidemic prevailing in Cobb county among the children. It was unlike anything he had seen or that was described in the books. It is a peculiar eruption on the body and extremities—not on the neck and face—accompanied with fever and sore throat. Had prevailed to a considerable extent at Acworth, and fatally. He had used chlorate potash, stimulating diaphoretics, gargles, etc.

Dr. Battey thinks a similar epidemic is prevailing in Alabama, North Georgia, and South Carolina. Had a letter from Cobb county asking advice in the same sort of cases. Had seen several cases in the city with papular eruption and sore throat, but no exudation on tonsils or the throat.

Dr. Rauschenberg has seen diphtheria and scarlatina epidemics, one disease following the other in the same case at the same time.

Dr. Armstrong saw a case, with Dr. Simpson, eight days ago. He had been recently in the country, where diphtheria was prevailing. Two days after his return home, was taken sick with high fever and sore throat. There was a decided exudation on tonsils, but no eruption.

Dr. Battey saw a case of supposed stye on the eye-lid; thought there was something unusual about its appearance, and, in consultation with Dr. Calhoun, discovered there was a diphtheritic exudation on the conjunctiva—something he had not seen before.

Dr. Rauschenberg thinks the same cause will produce the two diseases, diphtheria and scarlatina—diphtheria in one patient and scarlatina in another—owing to the peculiar physiological condition of the patient.

Dr. Woodhull had seen a good deal recently in the journals of malarial hæmaturia. Would like to ask if it did not occur almost entirely in the pine woods.

Dr. Connally had lived where malarial hæmaturia was prevalent at certain seasons of the year, and had treated it. The high, dry pine woods were almost exempt from it. On the water courses and ponds, and in the oaky woods, where the malaria was generated to the greatest extent, there we had hæmaturic fever in its worst form.

On motion, Academy adjourned.

---

A. W. CALHOUN, M.D., Reporter.

---

Atlanta, October 12, 1874.

Dr. V. H. Taliaferro in the Chair.

Dr. J. P. Logan was treating, with Dr. W. F. Westmoreland, a case of enlarged prostate gland, with 20 drops of fluid extract ergot and 10 drops of tincture belladonna, four times daily, with good results. In treating such affections, had usually had but little success, but with these remedies a marked effect was produced in reducing pain, and patient now feels himself well. This was an acute case of enlargement, the result of some exposure. This is but a single case, but the good derived is so decided that he is diposed to give some credence to the effect of the remedies.

Dr. Battey asked if other remedies had been unsuccessfully tried?

Dr. Logan replied that the case was of one week's duration, and quite stubborn under treatment by other remedies. Doesn't know positively the *rationale* of the action of the medicines, but is inclined to believe that the arterioles are contracted and the congestion in the inflamed parts thus overcome. Could not doubt, however, that the two combined remedies were efficacious.

Dr. Woodhull thought perhaps it was a plausible explanation that the ergot would have its effect more directly upon a diseased structure, as upon the prostate in this case.

Dr. W. F. Westmoreland had read conflicting reports of the use of ergot in this connection, in the different journals, some contending that its action was, in the majority of cases, well marked. The reports, however, had been very conflicting.

Dr. Cumming had a patient with ten hemorrhoidal tumors, which had bled freely and reduced the individual very much. Was called in for surgical treatment, but instead, applied pure carbolic acid to the ulcerated tumors, rubbing it in. It was very painful for ten or twelve days. In three weeks he could scarcely find a trace of the ulcers or tumors. Two months have passed, and the patient is now perfectly restored.

In reply to Dr. Westmoreland's question as to whether the carbolic acid was applied all over, Dr. Cumming replied, Over every part. It caused much swelling, and the parts turned white as after using any caustic.

Dr. W. F. Westmoreland reported the following case: He was called to see an infant in which he found an inperforate anus, with absence of entire rectum. He first saw the little patient twenty-four hours after its birth, and although there was frequent retching and vomiting, the distension of the abdomen was not very marked. In consultation with Drs. Johnson, Sterling, Battey, Griggs, and Kendrick, it was decided that an operation should be made at once, with the view of reaching the bowel. An incision was made in the median line, at the point where the anal opening should have been, an inch and a half or two inches in depth, in the direction of the normal location of the rectum. The finger introduced to the depth of the wound, and higher in the pelvis by an inch or more, by forcing up the soft parts, no distended bowel could be felt, nor could any rudiments of the rectum be detected. A trocar was then introduced through the incision, and to the depth of an inch and a half beyond the incision, when it was felt to have entered a cavity. Upon the removal of the trocar it was found to be the peritoneal cavity, and not the bowel. Two other punctures were made, differently directed, with like results. Further effort to reach the bowel through the incision was abandoned, and all interference deferred for the time.

It was proposed, twenty-four hours later, when the distension became greater, to reach the bowel through the abdominal walls. The parents of the child declined to permit further interference, and the little sufferer died the third day after the operation. After death, although denied a post-mortem examination, was permitted to examine the abdomen, when termination of the closed bowel, or cul-de-sac, was readily detected at the lower portion of the sigmoid flexure.

Dr. Battey was called three months ago to a lady suffering the nausea of pregnancy. In two previous pregnancies had suffered similarly, and was only relieved by miscarriage. Told her that in all probability some disease of the uterus existed, and upon examination, found ulceration of the os and cervix. He treated her for one month, and she seemed to be getting along very well. In his absence, one month since, she miscarried. In previous miscarriages, had not confined herself to bed, and got along quite well. Cautioned her to be careful. She got up, however, and had pain, and Drs. Taliaferro and Kendrick took measures to check the pelvic cellulitis, but unsuccessfully. An abscess formed. Examination per vaginam failed to locate the seat of the inflammation. Complained of pain along the course of the femoral artery of the left thigh, passing down to the calf of the leg. Three weeks later had hæmaturia and evidences of hemorrhage from the kidneys. There was congestion about the left kidney. On last Thursday morning was again called, and found her prostrated and scarcely conscious of his (Dr. B.'s) presence. Under the use of brandy, she revived. In reply to questions, she said that in rising she felt something break and all pain suddenly cease, and a "fainty feeling" come over her. On the following morning found pus passing from the bowels, having opened into the colon. She is now doing well. Had had cases which discharged into the kidney, and thus passed off.

The Academy then adjourned.

